# Clarifying the GDF15-LTL relationship: The role of medications, lifestyle, and morbidity

**DOI:** 10.1016/j.jnha.2025.100558

**Published:** 2025-04-14

**Authors:** Chia-Ter Chao

**Affiliations:** a Department of Internal Medicine, Min-Sheng General Hospital, Taoyuan City, Taiwan; b Division of Nephrology, Department of Internal Medicine, National Taiwan University Hospital and National Taiwan University College of Medicine, Taipei, Taiwan; c Graduate Institute of Toxicology, National Taiwan University College of Medicine, Taipei, Taiwan; d Graduate Institute of Medical Education and Bioethics, National Taiwan University College of Medicine, Taipei, Taiwan

Dear Editor,

I read with great interest the work of Yu et al. [1], which examined the association between serum growth differentiation factor 15 (GDF15), mitochondrial DNA copy number, and leukocyte telomere length (LTL) cross-sectionally in a well-maintained cohort. They demonstrated that GDF15 levels were inversely correlated with LTL, independent of demographic profile, various anthropometric and physical parameters, and lipid and glycemic status. Their findings are both important and thought provoking; however, several factors that may influence serum GDF15 levels should be considered and adjusted before interpreting the observed associations.

First, medications have been shown to affect serum GDF15 levels. Since 2019, the anti-diabetic drug metformin has been found to increase circulating GDF15 concentrations in both animals and humans, regardless of diabetes status, partially mediating metformin’s body weight reduction effect [2]. In Yu et al.’s study, the participants had a mean glycated hemoglobin (HbA1c) level of 6.2 ± 1.3 %, with higher HbA1c levels in those with greater GDF15 concentrations. This suggests that a proportion of these patients might have been receiving metformin for diabetic management. Moreover, several bitter-tasting medications and compounds may also alter serum GDF15 levels. In a cross-over study, Wang et al., demonstrated that oral hydroxychloroquine significantly increased plasma GDF15 levels in healthy volunteers, independent of food intake or obesity status [3]. Therefore, accounting for participants’ medication profiles, particularly the abovementioned ones, and adjusting for them accordingly would be prudent to enhance the interpretability of their study findings.

Another important consideration when investigating GDF15 levels is lifestyle and morbidity status. Although intra-individual variations in GDF15 levels are low, existing literature indicates that cardiac function (as reflected by B-type natriuretic peptide levels) and smoking significantly modulate serum GDF15 levels among healthy individuals [4]. Additionally, a longitudinal follow-up study revealed that morbidities affecting different organs (kidney, heart and liver) influenced changes in serum GDF15 levels over time, independent of age [5]. Given that changes in organ functions are known precipitators of LTL alterations over time, outlining the morbidity profiles of study participants would provide deeper insight into the association between GDF15 levels and LTL.

In conclusion, Yu et al.’s study provides valuable information regarding the relationship between GDF15 levels and LTL. However, considering the potential influences of medications, lifestyle factors, and morbidity status on GDF15 levels would strengthen the validity of their findings. A more comprehensive adjustment for these confounders could help elucidate the true nature of the observed associations, ensuring that the observed relationship is not driven by external factors.

## CRediT authorship contribution statement

CTC is solely responsible for drafting of this manuscript.

## Consent for publication

Not applicable

## Ethics, consent, and permission

Not applicable.

## Declaration of Generative AI and AI-assisted technologies in the writing process

I have not used any AI at all.

## Funding disclosure

None declared.

## Availability of data and material

Not applicable.

## Declaration of competing interest

The author reports no relationships that could be construed as a conflict of interest associated with this manuscript.
